# The Examination Rooms, Theatre, and Lavatories

**Published:** 1912-05-11

**Authors:** 


### THE IDEAL SCHOOL CLINIC.

### II.—The Examination Rooms, Theatre, and Lavatories.

THE wings of the clinic are occupied by the rooms devoted to the examination and treatment of patients. It is a moot point whether a demarcation between boy and girl patients is necessary; most authorities consider that there is no need for such a separation, and that the ordinary outpatient routine followed in children's hospitals may be employed in the clinic. Certainly there is no necessity to enforce separation in the waiting-room, though such distinction may be made in the examining and treating rooms, and must be made in the lavatories. Two working rooms (for a clinic such as we have in mind comparatively small sized rooms, 14 feet by 12 feet, are quite satisfactory) must be provided in each wing; those on the one side are used for surgical cases, those on the other for medical cases. Where no separate dental department is provided the dentist uses the surgical side on alternate days or in the afternoons, according to arrangement.

The rooms on the surgical side should consist of an examining room, which must be fitted with lamp for examining throat and ear cases, with a convenient sized examining couch, with the usual appliances for examining urine and doing quick clinical laboratory methods, with a table, chair, cabinet for examination instruments and apparatus, and with weighing machine and measuring rod. One or two tipping hand-basins with hot and cold water taps should be provided. The second room, opening from the examining room, is used as an operating room, and must be furnished accordingly. It will be borne in mind that very few operating cases that come to the clinic from the schools need a general anæsthetic (certainly not more than 2 per cent.), and there is no necessity, therefore, to provide a special anæsthetist's room. Apparatus for administering a general anæsthetic must always be provided, though it will not often be required. Cases of tonsils and adenoids are generally best done without any anæsthetic at all. A small recovery room, provided with ample lavatory accommodation, must be provided. The operating room requires a simple operating-table capable of being adjusted to two positions; a more elaborate model is not necessary nor desirable. In constructing the room all angles must be rounded, and the materials used for floor, wall, and ceiling should permit of easy and rapid cleansing.

Adequate provision must be made for natural and artificial lighting, while ventilation and heating must be carefully considered. In a small clinic open fireplaces, even for the operating theatre, are by far the best. In larger institutions radiators with low-pressure hot-water pipes seem to answer every purpose. Where the rooms are used by the dentist alternately with the surgeon—a convenient arrangement if the clinic does not serve too many contributing centres—proper provision must be made for his necessities in both the examining and the operating room. In such a case a dentist's chair, with the usual accompaniments, will be required.

Most of the surgical work done at the clinic will be of the character of that now done in the out-patient department of every children's hospital. That is to say, it will consist mainly of cases of tonsils and adenoids, discharging ears, excision of small cysts and tumours, simple incisions, nævi, deformities, and enlarged glands. An electric cautery, or, if it is preferred, a portable $CO_2$ apparatus for nævi is very useful. Enlarged glands, if they need excision, should be thoroughly extirpated—an operation of some magnitude which it is hardly justifiable to do in an out-patient department of the school clinic; in these cases, therefore, the child must be referred to a hospital. Deformities will have to be carefully treated. The most common are flat feet and lateral curvature. For the former, accurately modelled celluloid insets made from plaster casts of the foot are required; these may be made either in the examining or in the operating room, and should be manufactured by the surgeon himself or by a skilled nurse, whom he has himself instructed, under his immediate supervision. The lateral curvature cases are best treated by the crawl method, together with active movements under the modified Klapp method as recommended by Spitzy. These exercises can be performed on certain days in the central waiting-hall.

The medical examination and treatment rooms should be similar in size to the surgical, their furniture will be more simple, but everything must be provided that is required. An electric battery for galvanic treatment is very useful. The bath-room attached to the clinic should contain a shower or douche, as well as the usual bath. Here, as elsewhere, the usual requirements of a modern out-patient department should be remembered. Whatever is provided should be of first-rate quality so as to endure. The policy of buying cheap wares is most uneconomical both for school clinics and for hospitals. Ample lavatory accommodation, with hot and cold water taps, must be provided. A word of caution is necessary with regard to the water-closets. These must be of the usual hospital type, with full flush, but the seats must be much lower and smaller and—a very necessary precaution—the chain-pulls must be enclosed in tubes, so as to prevent any accidents.* As children very often leave the closets unflushed, the attendant should make a point of visiting the lavatories regularly.

It is better to engage a special clinic attendant than to entrust the charge of overseeing the housekeeping to the already overworked and underpaid school caretaker.

* This precaution is often overlooked in children's hospitals and in schools. There are cases on record where a child has been accidentally strangled by a loose chain pull.

